# Impact of arrhythmia on diagnostic performance of adenosine stress CMR in patients with suspected or known coronary artery disease

**DOI:** 10.1186/s12968-015-0195-0

**Published:** 2015-11-05

**Authors:** Simon Greulich, Hannah Steubing, Stefan Birkmeier, Stefan Grün, Kerstin Bentz, Udo Sechtem, Heiko Mahrholdt

**Affiliations:** Department of Cardiology, Robert Bosch Medical Center, Auerbachstrasse 110, 70376 Stuttgart, Germany

**Keywords:** CMR, Adenosine stress, Arrhythmia, Coronary artery disease, Risk stratification

## Abstract

**Background:**

The diagnostic performance of adenosine stress cardiovascular magnetic resonance (CMR) in patients with arrhythmias presenting for work-up of suspected or known CAD is largely unknown, since most CMR studies currently available exclude arrhythmic patients from analysis fearing gating problems, or other artifacts will impair image quality. The primary aim of our study was to evaluate the diagnostic performance of adenosine stress CMR for detection of significant coronary stenosis in patients with arrhythmia presenting for 1) work-up of suspected coronary artery disease (CAD), or 2) work-up of ischemia in known CAD.

**Methods:**

Patients with arrhythmia referred for work-up of suspected CAD or work-up of ischemia in known CAD undergoing adenosine stress CMR were included if they had coronary angiography within four weeks of CMR.

**Results:**

One hundred fifty-nine patients were included (*n* = 64 atrial fibrillation, *n* = 87 frequent ventricular extrasystoles, *n* = 8 frequent supraventricular extrasystoles). Of these, *n* = 72 had suspected CAD, and *n* = 87 had known CAD. Diagnostic accuracy of the adenosine stress CMR for detection of significant CAD was 73 % for the entire population (sensitivity 72 %, specificity 76 %). Diagnostic accuracy was 75 % (sensitivity 80 %, specificity 74 %) in patients with suspected CAD, and 74 % (sensitivity 71 %, specificity 79 %) in the group with known CAD. For different types of arrhythmia, diagnostic accuracy of CMR was 70 % in the atrial fibrillation group, and 79 % in patients with ventricular extrasystoles. On a per coronary territory analysis, diagnostic accuracy of CMR was 77 % for stenosis of the left and 82 % for stenosis of the right coronary artery.

**Conclusion:**

The present data demonstrates good diagnostic performance of adenosine stress CMR for detection of significant coronary stenosis in patients with arrhythmia presenting for work-up of suspected CAD, or work-up of ischemia in known CAD. This holds true for a per patient, as well as for a per coronary territory analysis.

## Background

Coronary artery disease (CAD) is the leading cause of death in the western world [1]. Current clinical practice guidelines recommend noninvasive stress testing for 1) work-up of suspected CAD [2], and 2) work-up of ischemia in known CAD [3].

Adenosine stress cardiovascular magnetic resonance (CMR) is a noninvasive stress-testing modality offering high diagnostic accuracy without need for radiation or acoustic window [4–7]. However, up to 90 % of patients with CAD suffer from frequent ventricular ectopic beats [8], and/or other arrhythmias [9, 10]. Unfortunately, the diagnostic performance of adenosine stress CMR in this important subgroup is widely unknown, since most CMR studies currently available exclude arrhythmic patients from analysis [6, 11–13] fearing gating problems, or other artifacts will impair image quality [14].

Thus, our primary aim was to evaluate the diagnostic performance of adenosine stress CMR for detection of significant coronary stenosis in patients with arrhythmia presenting for 1) work-up of suspected CAD, or 2) work-up of ischemia in known CAD. In addition, we aimed to assess the diagnostic performance of adenosine stress CMR for different types of arrhythmia and for different coronary territories.

## Methods

### Patient population

**Fig. 1:**
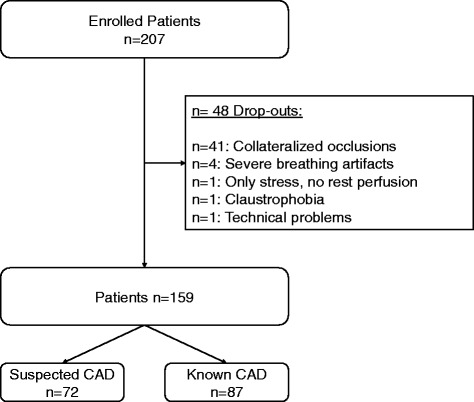
Flow chart demonstrating the study population

All patients referred for 1) work-up of suspected CAD, and 2) work-up of ischemia in known CAD undergoing adenosine stress CMR at our institution between January 2011 and June 2014 were prospectively screened for study enrolment on a consecutive basis. We included all patients with arrhythmia during the stress CMR procedure (see definition of arrhythmia below), who underwent invasive coronary angiography within 4 weeks before or after the stress CMR, and who gave written informed consent to the protocol, which was approved by the local institutional review board (University of Tübingen, Germany). Exclusion criteria for the analysis were collateralized total occlusions revealed by coronary angiography, since in those cases no ischemia may be present despite occlusion, non-diagnostic images due to breathing artifacts, withdrawal of consent before completion of procedure, or other technical problems (Fig. 1). Baseline characteristics of the patient population can be viewed in Table 1.

**Table 1:** Baseline characteristics

	Entire group	Suspected CAD	Known CAD	p
	(*n* = 159)	(*n* = 72)	(*n* = 87)	
Age (yrs)	71.1 ± 10	69.9 ± 10.4	72.2 ± 9.6	0.17
Gender (female)	55 (35 %)	39 (54 %)	16 (18 %)	<0.05
CAD Risk Factors				
Diabetes	51 (32 %)	20 (28 %)	31 (36 %)	0.31
Hypertension	127 (80 %)	58 (81 %)	69 (79 %)	1
Smoking^a^	49 (31 %)	18 (25 %)	31 (36 %)	0.17
Hyperlipidemia	105 (66 %)	39 (54 %)	66 (76 %)	0.004
Family history of CVD	55 (35 %)	24 (33 %)	31 (36 %)	0.74
Menopause^b^	52 (95 %)^b^	37 (95 %)^b^	15 (94 %)^b^	1
Obesity (BMI ≥ 30 kg/m^2^)	30 (19 %)	17 (24 %)	13 (15 %)	0.15
Number of risk factors	2.9 ± 1.2	2.9 ± 1.3	3.0 ± 1.2	0.53
Cardiac Arrhythmia				
Heart rate at rest (beats/min.)	67 [60–78]	68 [60–80]	66 [59–78]	0.71
Heart rate at stress (beats/min.)	85 [77–99]	85 [78–101]	85 [77–98]	0.89
Atrial fibrillation	64 (40 %)	32 (44 %)	32 (37 %)	0.34
VES	87 (55 %)	35 (49 %)	52 (60 %)	0.20
Couplets	15 (9 %)	4 (6 %)	11 (13 %)	0.18
Triplets	6 (4 %)	2 (3 %)	4 (5 %)	0.69
Bigeminus	32 (20 %)	12 (17 %)	20 (23 %)	0.43
Trigeminus	6 (4 %)	3 (4 %)	3 (3 %)	1
SVES	8 (5 %)	5 (7 %)	3 (3 %)	0.47
Medication				
Statins	92 (58 %)	34 (47 %)	58 (67 %)	0.03
Beta-blockers	100 (63 %)	38 (53 %)	62 (71 %)	0.03
Aspirin	93 (59 %)	34 (47 %)	59 (68 %)	0.02
ARB	105 (66 %)	41 (57 %)	64 (74 %)	0.06
Nitrates	37 (23 %)	12 (17 %)	25 (29 %)	0.13
Diuretics	77 (48 %)	31 (43 %)	46 (53 %)	0.33
Symptoms (multiple possible)				
Chest pain	107 (67 %)	43 (60 %)	64 (74 %)	0.43
Dyspnea	87 (55 %)	46 (64 %)	41 (47 %)	0.04
Palpitations	16 (10 %)	9 (13 %)	7 (8 %)	0.09
Syncope	10 (6 %)	5 (7 %)	5 (6 %)	0.75
Reduced LV-EF	56 (35 %)	23 (32 %)	33 (38 %)	0.61
ECG abnormality	105 (66 %)	37 (51 %)	68 (78 %)	<0.001
Wall motion abnormality	46 (29 %)	12 (17 %)	34 (39 %)	0.02

Values are n (%), mean ± SD or median [IQR]

*suspected CAD* CMR work-up of suspected CAD in patients without history of CAD, *known CAD* CMR work-up of ischemia in patients with prior myocardial infarction and/or revascularization procedure (PCI or CABG), *CAD* coronary artery disease, *PCI* percutaneous coronary intervention, *CABG* coronary artery bypass graft, *CVD* cardiovascular disease, *BMI* body mass index, *VES* ventricular extrasystoles, *SVES* supraventricular extrasystoles, *ARB* angiotensin receptor blocker, *CMR* cardiac magnetic resonance, *LV-EF* left ventricular ejection fraction, *ECG* electrocardiography

^a^Current or ever-smokers

^b^Calculated for females

### Definitions

Relevant coronary stenosis/CAD was defined as ≥70 % narrowing of the luminal diameter in at least one projection of at least one major epicardial artery, or ≥50 % narrowing of the left main [2].

Suspected CAD: Patients without prior history of CAD.

Known CAD: Patients with prior myocardial infarction and/or revascularization procedure(s) such as percutaneous coronary intervention (PCI) or coronary artery bypass graft (CABG).

CAD-type late gadolinium enhancement (LGE): Subendocardial or transmural LGE consistent with prior myocardial infarction [15].

Arrhythmia was defined as atrial fibrillation, and/or frequent ectopic beats >20/min (of ventricular or supraventricular origin) [13]. All arrhythmias were detected by ECG and/or Holter ECG, and had to be present during both adenosine stress and rest perfusion.

Analysis per coronary territory was made on a 17 segment model basis according to AHA guidelines as previously described [16]. Left coronary artery (LCA) included the following coronary arteries: left main (LM), left anterior descending (LAD) and left circumflex artery (LCX).

### CMR protocol

Electrocardiogram (ECG) gated CMR imaging was performed in breath-hold using a 1.5 T Magnetom Aera (Siemens-Healthcare, Germany) in line with recommendations of the Society of Cardiac Magnetic Resonance, and the European Society of Cardiology Working Group EuroCMR, respectively [17].

Details of the CMR protocol have been reported previously [18]. In brief, steady-state free-precession cine images for assessment of LV function were acquired in multiple short-axis (every 10 mm throughout the LV) and three long-axis views. Adenosine (140 μg · kg^−1^ · min^−1^) was infused under continuous electrocardiography and blood pressure monitoring for approximately 3 min. At 2.5 min into the infusion, gadolinium (0.07 mmol/kg Gadodiamide or Gadopentetate-Dimeglumine) first-pass imaging for assessment of stress perfusion was performed in three short axis views (basal, mid, apical, matched to cine locations excluding most basal and apical slices) using a saturation-recovery, single-shot, gradient-echo sequence (90° pre-pulse before each slice; echo time, 1.1 ms; delay time, 85–100 ms; temporal resolution, 110–125 ms; voxel size, 3.0 × 1.8 × 8.0 mm). In order to speed up imaging parallel imaging with 2-fold acceleration was employed. Repeat first-pass images without adenosine 15 min later were performed for assessment of rest perfusion. Five minutes after rest perfusion (additional 0.07 mmol/kg Gadodiamide or Gadopentetate-Dimeglumine), late gadolinium enhancement was performed using a segmented inversion-recovery technique in the identical views as cine-CMR. The image acquisition protocol was completed in about 45 min.

### CMR analysis

Scans were analyzed by consensus of two experienced observers (S.G., H.M.), who were blinded to patient identity, clinical information, and the angiography results. A perfusion defect was defined as a regional dark area, that 1) persisted for >2 beats while other regions enhanced during the first-pass of contrast through the myocardium, and 2) involved the subendocardium [19, 20]. Dark rim artifact was not regarded as perfusion deficit using previously described criteria [21].

Cine and contrast images were evaluated as described elsewhere [22]. In brief, endocardial and epicardial borders were outlined on the short axis cine images. Volumes and ejection fraction were derived by summation of epicardial and endocardial contours.

In patients referred for work-up of suspected CAD the Duke algorithm was used for diagnosis of CAD [18]: Patients were diagnosed having relevant stenosis/CAD if they had 1) evidence of LGE consistent with a prior myocardial infarction, or 2) no evidence of prior myocardial infarction, but perfusion defects present with adenosine that were absent or reduced at rest (reversible perfusion defect). If patients showed a matched perfusion defect under stress and rest perfusion, patients were considered having no relevant stenosis/CAD [18].

In patients referred for work-up of ischemia in known CAD two different algorithms were used depending on the presence of ischemic scar: 1) In the absence of ischemic scar on LGE images relevant stenosis/ischemia was defined as the presence of a reversible perfusion defect as described above [18]. 2) In the presence of ischemic scar on LGE images ischemia was defined as a mismatch between the first-pass stress perfusion defect and enhancement seen on LGE sequences, whereas a match between the first-pass stress perfusion defect and LGE was considered as chronic myocardial infarction with no additional reversible ischemia [23].

### Coronary angiography and analysis by coronary artery territory

Coronary angiography was performed by standard techniques [24] and analyzed masked to identity, clinical information, and CMR results. Significant CAD was defined as ≥70 % narrowing of the luminal diameter in at least one projection of at least one major epicardial artery, or ≥50 % narrowing of the left main [2]. Native vessels with a diameter smaller than 2 mm were excluded from the analysis. Two experienced interventional cardiologists (S.G, H.M.) blinded to the results of the CMR imaging visually evaluated the coronary angiograms by consensus.

### Statistical analysis

Absolute numbers and percentages were computed to describe the patient population. All continuous variables were tested for normality. Normally distributed continuous variables were expressed as means (with standard deviation) and skewed variables were presented as medians (with quartiles). Comparisons between groups were made using the Mann-Whitney *U* test or the Fisher’s exact test, as appropriate. Statistical tests were two-tailed; *p*-value < 0.05 was considered significant. Positive and negative likelihood ratios (LR) were calculated. All statistical analyses were performed using SPSS, version 22.0 (IBM Corp., Armonk, NY, USA).

## Results

### Patient characteristics

In total 159 patients were included in the final analysis (Fig. 1). Table 1 summarizes the patient characteristics. At inclusion, patients were 71 ± 10 years of age and predominantly male (65 %). Atrial fibrillation was present in 64 patients (40 %), 87 patients (55 %) suffered from frequent ventricular extrasystoles (VES), and 8 patients (5 %) showed frequent supraventricular extrasystoles (SVES). The majority (67 %) had chest pain as primary reason to suspect significant CAD, followed by dyspnea (55 %), and palpitations (10 %).

Seventy-two patients were referred for work-up of suspected CAD, and 87 patients were referred for work-up of ischemia in known CAD. The group with known CAD was older (72.2 ± 9.6) with fewer females (18 %) than the group with suspected CAD (age 69.9 ± 10.4, *p* = 0.17; 54 % females, *p* < 0.05). Patients presenting with known CAD had a higher prevalence of hyperlipidemia (*p* = 0.004), and wall motion abnormalities (*p* = 0.02) than patients with suspected CAD. Of note, the prevalence of the different types of arrhythmia was similar between the two groups.

### General CMR findings

General CMR results are displayed in Table 2. Left ventricular ejection fraction (LV-EF) in the study population was mildly impaired (median 54 %) with normal mean cardiac volumes. Overall, CMR perfusion revealed LCA ischemia in 31 %, and RCA ischemia in 23 % of patients. CAD-type LGE was present in 47 % of patients.

**Table 2:** CMR results

Parameter	Entire group	Suspected CAD	Known CAD	p
	(*n* = 159)	(*n* = 72)	(*n* = 87)	
LV-EF (%)	54 [39–66]	61 [45–67]	50 [34–64]	0.04
LV-EDV (ml)	124 [101–168]	116 [91–145]	133 [106–181]	0.01
LV-ESV (ml)	54 [32–98]	49 [28–69]	68 [40–109]	0.007
LA (cm^2^)	26 [21–35]	27 [22–38]	26 [21–34]	0.43
IVS (mm)	12 [10–13]	11 [10–13]	12 [10–14]	0.58
Ischemia LCA	49 (31 %)	13 (18 %)	36 (41 %)	0.001
Ischemia RCA	36 (23 %)	7 (10 %)	29 (33 %)	<0.001
CAD-type LGE	74 (47 %)	15 (21 %)	59 (68 %)	<0.001

Values are median [IQR], or n (%)

*CAD* coronary artery disease, *suspected CAD* CMR work-up of suspected CAD in patients without history of CAD, *known CAD* CMR work-up of ischemia in patients with prior myocardial infarction and/or revascularization procedure (PCI or CABG), *LV* left ventricular, *EF* ejection fraction, *EDV* end-diastolic volume, *ESV* end-systolic volume, *LA* left atrium, *IVS* interventricular septum, *LCA* LM + LAD + LCX, *LCA* left coronary artery, *LM* left main, *LAD* left anterior descending, *LCX* left circumflex artery, *LGE* late gadolinium enhancement

Among patients with known CAD, LV-EF was significantly lower compared to the suspected CAD group, *p* = 0.04. Conversely, left ventricular end-diastolic volumes (LV-EDV) and left ventricular end-systolic volumes (LV-ESV) were significantly larger in the known CAD group (LV-EDV *p* = 0.01, LV-ESV *p* = 0.007 respectively). In addition, patients with known CAD were diagnosed with relevant stenosis/ischemia more frequently than patients with suspected CAD (*p* = 0.001, *p* < 0.001, respectively). CAD-type LGE was also more common in the known CAD group (68 vs. 21 %, *p* < 0.001).

### Diagnostic performance of CMR

#### Entire population

Overall diagnostic accuracy of adenosine stress CMR for the detection of ≥70 % stenosis on coronary angiography was 73 % (sensitivity 72 %, specificity 76 %) for patients with suspected or known CAD (Table 3). On a per coronary territory basis the diagnostic accuracy of CMR was 77 % for LCA stenosis (sensitivity 78 %, specificity 77 %) and 82 % for detection of RCA stenosis (sensitivity 63 %, specificity 88 %).

**Table 3:** Diagnostic performance of CMR stress testing for the detection of ≥70 % stenosis on coronary angiography in all patients (*n* = 159)

	Per patient	LCA^a^	RCA
All Types of Arrhythmia^b^			
Sensitivity	72 % (49/68)	78 % (53/68)	63 % (24/38)
Specificity	76 % (69/91)	77 % (70/91)	88 % (107/121)
Diagnostic Accuracy	73 % (118/159)	77 % (123/159)	82 % (131/159)
LR+	3.00	3.39	5.25
LR-	0.37	0.29	0.42
AFib Only			
Sensitivity	71 % (25/35)	81 % (22/27)	63 % (10/16)
Specificity	69 % (20/29)	76 % (28/37)	88 % (42/48)
Diagnostic Accuracy	70 % (45/64)	78 % (50/64)	81 % (52/64)
LR+	2.29	3.38	5.25
LR-	0.42	0.25	0.42
VES Only			
Sensitivity	74 % (23/31)	75 % (30/40)	65 % (13/20)
Specificity	82 % (46/56)	81 % (38/47)	90 % (61/67)
Diagnostic Accuracy	79 % (69/87)	78 % (68/87)	85 % (74/87)
LR+	4.11	3.95	6.05
LR-	0.32	0.31	0.39

Values are % (n)

*AFib* atrial fibrillation, *VES* ventricular extrasystoles, *SVES* supraventricular extrasystoles, *LR+* positive likelihood ratio, *LR-* negative likelihood ratio

^a^LCA = LM + LAD + LCX, abbreviations see Table 2

^b^All types of arrhythmia: *n* = 64 AFib + *n* = 87 VES + *n* = 8 SVES

Looking at patients presenting with atrial fibrillation (*n* = 64) revealed a diagnostic accuracy of 70 % for CMR (sensitivity 71 %, specificity 69 %), which is lower than in the 87 patients presenting with VES (diagnostic accuracy 79 %, sensitivity 74 %, specificity 82 %). On a per coronary territory basis, the diagnostic accuracy for detection of LCA and RCA stenosis was good in patients with atrial fibrillation (78 %, 81 % respectively), and in patients with VES (78 %, 85 % respectively).

Considering the low number of patients with SVES (*n* = 8), these patients were included in the entire population analysis. Five of those patients were in the suspected CAD group, and three out of five were classified correctly as negative by CMR. The other three patients had known CAD, two of them had coronary stenosis on coronary angiography, one of them was correctly identified by CMR.

### Patients with suspected CAD

Diagnostic accuracy of CMR stress testing for the detection of ≥70 % stenosis in patients with suspected CAD was 75 % (sensitivity 80 %, specificity 74 %), positive likelihood ratio (LR) 3.08, negative LR 0.27 (Table 4). The prevalence of significant coronary stenosis on coronary angiography was 14 % (10 out of 72 patients with suspected CAD).

**Table 4:** Diagnostic performance of CMR stress testing for the detection of ≥70 % stenosis on coronary angiography in patients with suspected CAD by use of the Duke algorithm^a^

	Per patient	LCA^b^	RCA
Sensitivity	80 % (8/10)	80 % (8/10)	100 % (1/1)
Specificity	74 % (46/62)	76 % (47/62)	89 % (63/71)
Diagnostic Accuracy	75 % (54/72)	76 % (55/72)	89 % (64/72)
LR+	3.08	3.33	8.33
LR-	0.27	0.26	0

Values are % (n)

*suspected CAD* CMR work-up of suspected CAD in patients without history of CAD

^a^Presence of CAD-type LGE or stress induced perfusion defect

^b^LCA = LM + LAD + LCX; n = 32 AFib + n = 35 VES + *n* = 5 SVES; other abbreviations see Table 3

**Fig. 2:**
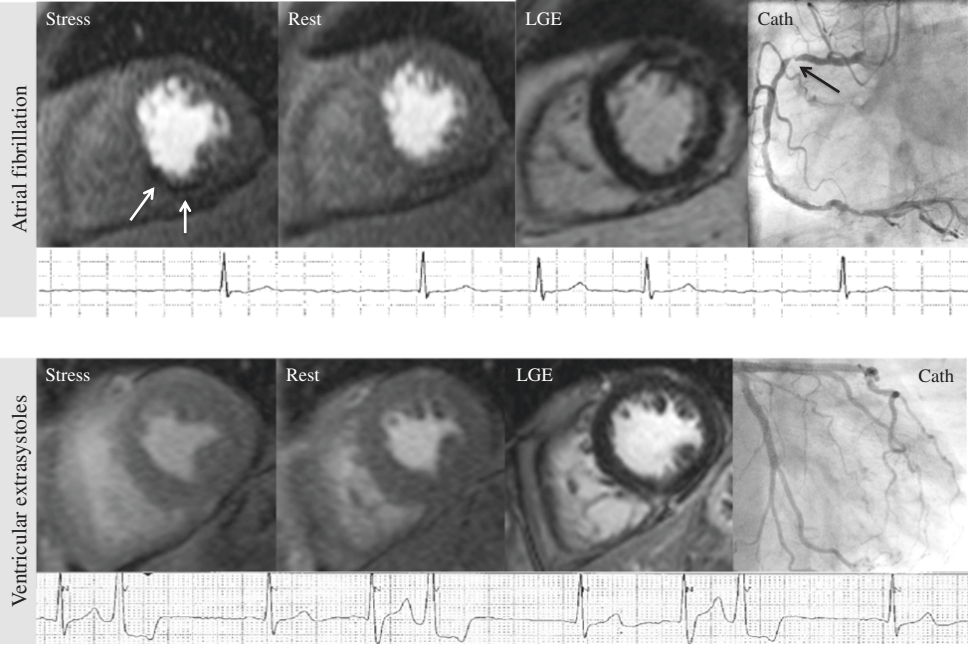
Patients with suspected CAD, but different types of arrhythmia: *Top row*: 72-year old male with atrial fibrillation presenting for work-up of suspected CAD. CMR revealed no LGE, but a reversible perfusion defect at the inferoseptal wall (*white arrows*), highly suggestive of significant RCA stenosis. Coronary angiography revealed high-grade RCA stenosis (*black arrow*). *Bottom row*: 69-year old female with frequent VES and atypical chest pain presenting for work-up of suspected CAD. LGE was negative, stress perfusion revealed no perfusion defect, resulting in the CMR diagnosis “no CAD”. Coronary angiography confirmed unobstructed coronary arteries

CMR identified 80 % of the patients with suspected CAD and stenosis of the LCA correctly, yielding to a diagnostic accuracy of 76 % (specificity 76 %), positive LR 3.33 and negative LR 0.26. For the RCA, CMR revealed a diagnostic accuracy of 89 %, with a sensitivity of 100 %, and a specificity of 89 %, positive LR 8.33, negative LR 0. Figure 2 demonstrates two typical patients with suspected CAD and different types of arrhythmia.

### Patients with known CAD

The diagnostic accuracy of CMR stress for detection of ≥70 % stenosis in patients with known CAD was 74 % (sensitivity 71 %, specificity 79 %), positive LR 3.38, negative LR 0.37, see Table 5. The prevalence of significant coronary stenosis on coronary angiography was 67 % (58 out of 87 patients with known CAD).

**Table 5:** Diagnostic performance of CMR stress testing for the detection of ≥70 % stenosis on coronary angiography in patients with known CAD

	Per patient	LCA^a^	RCA
Sensitivity	71 % (41/58)	78 % (45/58)	62 % (23/37)
Specificity	79 % (23/29)	79 % (23/29)	88 % (44/50)
Diagnostic Accuracy	74 % (64/87)	78 % (68/87)	77 % (67/87)
LR+	3.38	3.71	5.17
LR-	0.37	0.28	0.43

Values are % (n)

*known CAD* CMR work-up of ischemia in patients with prior myocardial infarction and/or revascularization procedure (PCI or CABG)

^a^LCA = LM + LAD + LCX, *n* = 32 AFib + *n* = 52 VES + *n* = 3 SVES, abbreviations see Table 3

**Fig. 3:**
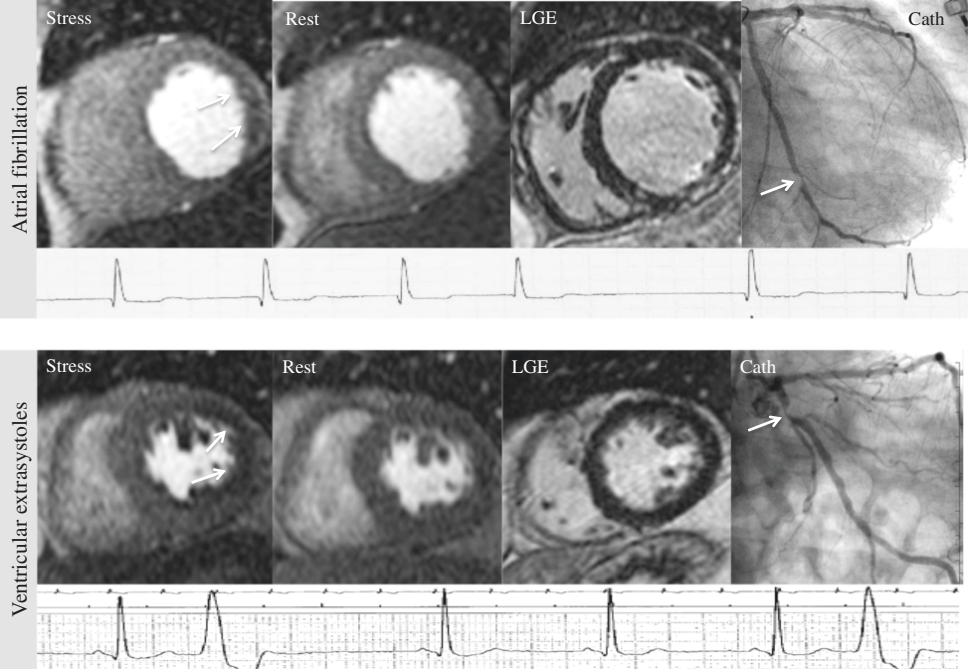
Patients with known CAD. *Top row*: 71-year old female with atrial fibrillation and known CAD (myocardial infarction two years ago) presented for work-up of new ischemia. LGE revealed a transmural infarction of the inferior wall. Stress perfusion demonstrated a reversible perfusion defect of the lateral wall (*white arrows*), highly suggestive of significant LCX stenosis. This could be confirmed by coronary angiography: LCX had a high-grade proximal stenosis (*white arrow*), RCA showed coronary plaques, but no significant stenosis. *Bottom row*: 73-year old male with typical angina, frequent VES, and known CAD (prior stenosis of the LAD, in which PCI was performed 12 years ago). LGE revealed no scar, but stress perfusion demonstrated a large perfusion defect in the lateral wall, suggestive of LCX stenosis. On coronary angiography, severe LCX stenosis could be confirmed

CMR identified 78 % of patients with known CAD and stenosis ≥70 % in the LCA correctly, yielding a diagnostic accuracy of 78 % and a specificity of 79 %, positive LR 3.71, and negative LR 0.28. For the RCA, CMR revealed a diagnostic accuracy of 77 %, with a sensitivity of 62 %, and a specificity of 88 %, positive LR 5.17, negative LR 0.43. Typical CMR results are displayed in Fig. 3

## Discussion

To the best of our knowledge, this is the first study evaluating the diagnostic performance of adenosine stress CMR for detection of significant coronary stenosis in patients with different types of arrhythmia. Our data indicate that adenosine stress CMR performs well for detection of relevant coronary stenosis in patients with suspected CAD (diagnostic accuracy 75 %), and also in patients with known CAD (diagnostic accuracy 74 %), despite the presence of various arrhythmias during the CMR procedure. These results underscore the increasing value of adenosine stress CMR in the real world clinical routine.

### Patient characteristics

The average patient age and gender distribution are similar to previous stress CMR studies in which patients with arrhythmia where usually excluded from analysis [5]. This also holds true for the clinical symptoms leading to CMR referral in the present population [25]. Types of arrhythmia found in our patients include atrial fibrillation, frequent VES and frequent SVES, which are known to be associated with CAD [8–10]. As to expect, the subgroup with known CAD was older (72.2 ± 9.6 years), with fewer females (18 %) and higher prevalence of hyperlipidemia than the group with suspected CAD (69.9 ± 10.4 years, *p* = 0.17; 54 % females, *p* < 0.05).

### General CMR findings

Median LV-EF of all patients was 54 %, which is comparable to other studies evaluating the diagnostic performance of adenosine stress CMR in a mixed patient population comprising patients with suspected and known CAD [23]. Patients with known CAD had a lower LV-EF and larger end-diastolic and end-systolic volumes, most likely explained by the higher prevalence of ischemic scar represented by CAD-type LGE (68 vs. 21 % in the suspected CAD group), resulting in reduced LV-EF and ventricular remodeling. Ischemia was also more common in patients with known CAD than in the group with suspected CAD, respectively.

### Diagnostic performance of CMR

#### Entire population

Looking at the entire population, the diagnostic accuracy of stress CMR for the detection of ≥70 % stenosis on coronary angiography was 73 % (sensitivity 72 %, specificity 76 %) for all 159 patients with suspected or known CAD, and different types of arrhythmia. This is lower than reported in a large meta-analysis [5] calculating a sensitivity of 90 % and a specificity of 81 %. However, many studies of this meta-analysis excluded patients with arrhythmia to improve image quality, which most likely explains the difference to our data based on patients presenting with arrhythmia only.

Analyzing our entire population data per coronary territory demonstrate CMR to yield a diagnostic accuracy of 77 % for the LCA (sensitivity 78 %, specificity 77 %), and of 82 % for the RCA (sensitivity 63 %, specificity 88 %). This is also lower than in the meta-analysis mentioned above, demonstrating sensitivities of 83, 76 and 78 % and specificities of 83, 87, and 87 % for LAD, LCX and RCA, respectively. However, patients with arrhythmias were excluded in most studies of this meta-analysis [5].

Comparing patients with atrial fibrillation to patients with VES (Table 3) reveals a good diagnostic accuracy for LCA and RCA (78 and 81 %) in the atrial fibrillation group, and in patients with VES (78 and 85 %). However, the sensitivities for detecting relevant RCA stenosis were lower in both groups when compared to the LCA (atrial fibrillation 63 vs. 81 %, VES 65 vs. 75 %). This is in accordance with other studies [5], reporting a higher sensitivity for detection of stenosis in the LAD when compared to LCX and RCA, most likely due to the surface radiofrequency coil receiving lower signal intensities from the inferior and lateral segments [5].

#### Patients with suspected CAD

Evaluating the subgroup presenting for work-up of suspected CAD the diagnostic accuracy was 75 % (sensitivity 80 %, specificity 74 %). This is less than reported by Klem et al. [18], who first described the Duke algorithm for work-up of suspected CAD by combining LGE and perfusion sequences in 92 patients. However, in contrast to the present data, the Klem population only included one patient with atrial fibrillation and two patients with VES.

Among our 72 patients with suspected CAD the prevalence of significant coronary stenosis was low (14 %), underscoring the need of noninvasive imaging in patients with arrhythmia before undergoing coronary angiography. One reason for the low prevalence of CAD might be that patients with arrhythmia are at an “increased risk” to be referred to coronary angiography due to previous inconclusive exercise tests, or the presence of arrhythmia itself in combination with risk factors and complaints. Interestingly, this finding nicely matches the results of Smit et al. who performed a myocardial perfusion single photon emission computed tomography (SPECT) analysis in patients with atrial fibrillation and suspected CAD. The prevalence of CAD (≥70 % stenosis) in this group was 13 % [26].

In a per coronary territory analysis, the diagnostic accuracy for the LCA was 76 % (sensitivity 80 %, specificity 76 %). For the RCA our data revealed a diagnostic accuracy of 89 % (sensitivity 100 %, specificity 89 %), completely in line with the results of Klem et al. [18]. However, it must be noted that only one patient with suspected CAD had ≥70 % RCA stenosis.

#### Patients with known CAD

Focusing on the subgroup with known CAD the diagnostic accuracy of CMR stress testing for the detection of ≥70 % stenosis was 74 % (sensitivity 71 %, specificity 79 %). A study by Klein et al. investigated the diagnostic performance of adenosine perfusion CMR in 78 patients [27] reporting a diagnostic accuracy of 82 % for detection of ≥50 % stenosis (sensitivity 77 %, specificity 90 %), excluding patients with atrial fibrillation.

**Fig. 4:**
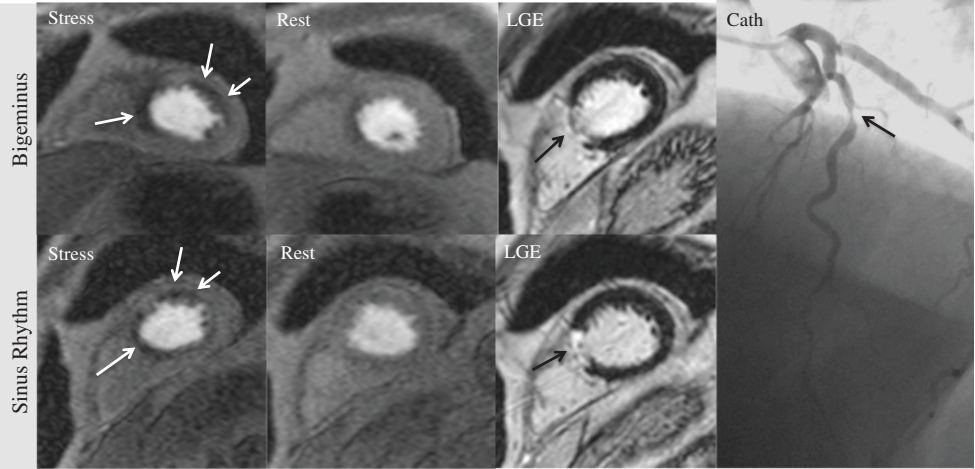
Two CMR exams with two different rhythms in one and the same patient. 77-year old male undergoing stress CMR two times within four weeks due to LAD in-stent restenosis early after intervention. One scan was performed during bigeminus (*upper row*), whereas the second scan was performed in sinus rhythm (*bottom row*). Note that the stress perfusion defect in the LAD territory (*left column*) could be detected in sinus rhythm, as well as during bigeminus

Per coronary territory the diagnostic accuracy for the LCA was 78 and 77 % for the RCA, which is also quite in line with other reports [23, 27]. Figure 4 demonstrates the feasibility of adenosine stress CMR in a patient presenting with frequent VES (bigeminus).

#### Limitations

Limitations of the present study are, that adenosine stress CMR was compared with invasive coronary angiography, which is not the perfect gold standard for comparison as functional significance of coronary obstruction and luminal diameter stenosis are known to show only moderate correlation. Furthermore, it is important to keep in mind that the algorithm used for CMR analysis of patients with suspected CAD is intended to detect significant obstruction of the epicardial coronaries compared to invasive coronary angiography (>70 % stenosis). Thus, perfusion defects that were considered as artifacts according to the algorithm used in this analysis may be a surrogate parameter for microvascular dysfunction. Hence, it might be possible that these patients who suffer partly from distinct anginal symptoms were classified as healthy by CMR, which could be confirmed by coronary angiography. Another limitation is the selection bias introduced by excluding patients with collateralized occlusions and CMR studies with severe breathing artifacts. Despite introducing a bias, we believe that the exclusion of collateralized occlusions is favorable in order to keep the data consistent, since in those cases no ischemia may be present despite occlusion of the vessel. Furthermore, removal of CMR data sets due to severe breathing artifacts seems reasonable since aim of our study was to evaluate the impact of arrhythmia (and not of severe breathing artifacts) on the diagnostic accuracy of an adenosine stress CMR test. Moreover, it should be stated that only a few patients (4 out of 163 patients, =0.02 %) were excluded due to severe breathing artifacts.

#### Clinical implications

On the basis of the data presented it may be safe to assume that the diagnostic performance of adenosine stress CMR for detection of significant coronary stenosis is somewhat impaired in patients presenting with arrhythmias compared to patients without arrhythmias (75 vs. 88 % for suspected CAD [18] and 74 vs. 82 % in known CAD [27]), but still sufficient for clinical routine use. In fact, with a sensitivity of 72 %, and specificity of 76 % (overall diagnostic accuracy of 73 %) adenosine stress CMR in patients with arrhythmias performs well in comparison to other stress testing modalities (SPECT: sensitivity 73–92 %, specificity 63–87; stress echocardiography: sensitivity 80–85 %, specificity 80–88 %; exercise ECG: sensitivity 45–50 %, specificity 85–90 %) [2], underscoring the value of adenosine stress CMR in a real world clinical setting.

Unfortunately, the current study was not designed to evaluate the prognostic value of stress CMR in patients with arrhythmias. However, given the encouraging results with regard to the diagnostic performance in those patients, and the results of the EuroCMR Registry demonstrating a good prognostic value of adenosine stress CMR in patients presenting for work-up of suspected CAD [28], including those with arrhythmias, it is likely that adenosine stress CMR also has a good prognostic value in arrhythmic patients. Nevertheless, additional data is needed to underscore this important point.

## Conclusions

The present data demonstrates a good diagnostic performance of adenosine stress CMR for detection of significant coronary stenosis in patients with arrhythmia presenting for 1) work-up of suspected CAD, or 2) work-up of ischemia in known CAD. This holds true in the entire population for a per patient, as well as a per coronary territory analysis.

## Abbreviations

AFib: Atrial fibrillation
CABG: Coronary artery bypass graft
CAD: Coronary artery disease
CMR: Cardiovascular magnetic resonance
ECG: Electrocardiogram
IQR: Interquartile range
LA: Left atrial
LCA: Left coronary artery
LCX: Left circumflex artery
LGE: Late gadolinium enhancement
LR: Likelihood ratio
LV: Left ventricle
LV-EDV: Left ventricular end-diastolic volume
LV-ESV: Left ventricular end-systolic volume
LV-EF: Left ventricular ejection fraction
PCI: Percutaneous coronary intervention
RCA: Right coronary artery
SPECT: Single photon emission computed tomography
SVES: Supraventricular extrasystoles
VES: Ventricular extrasystoles
